# Post-operative Hemoglobin Trends for Patients Undergoing Bariatric Surgery: A Retrospective Observational Study

**DOI:** 10.1007/s11695-026-08875-z

**Published:** 2026-07-25

**Authors:** Kate V. Lauer, Jamie Ho, Lily N. Stalter, Mads Owen, Andre Acra, Megha Brahmbhatt, David A. Harris, Anne O. Lidor, Luke M. Funk

**Affiliations:** 1 Department of Surgery, University of Wisconsin–Madison, Madison, USA; 2 University of Wisconsin–Madison, Madison, USA; 3 Department of Surgery, William S. Middleton Memorial Veterans Hospital, Madison, USA

**Keywords:** Bariatric surgery, Post-operative bleeding, Hemoglobin trends, Same day surgery

## Abstract

**Background:**

Prevalence of same day bariatric surgery programs is rising. While observational studies suggest same day outcomes are similar to inpatient programs, the ability to detect early post-operative bleeding in same day patients is uncertain. We compared hemoglobin values between patients who did or did not develop post-operative bleeding to delineate when hemoglobin trends diverge.

**Methods:**

Patients who underwent sleeve gastrectomy or gastric bypass at a single center from 2020 to 2024 were stratified into those who did or did not develop bleeding that required transfusion and/or surgical or endoscopic intervention. Three standardized hemoglobin draws (pre-operative, four-hour post-operative, morning of post-operative day 1) were compared between groups. Time to the first clinical sign of bleeding and intervention were evaluated for patients who developed clinically significant bleeding.

**Results:**

1,457 patients were included (mean [SD] age 45.4 [10.8] years; 85.5% White; 82.0% female, 60.6% sleeve gastrectomy). Thirty-nine patients (2.7%) developed bleeding. Mean pre-operative and four-hour post-operative hemoglobin values were similar. Hemoglobin values on the morning of post-operative day 1 were significantly lower for the patients who bled [10.7 g/dL (SD 1.6) vs. 12.5 g/dL (1.1); *p* < 0.001]. The median time to the first clinical sign of bleeding was 9 h (IQR 6.25–17.5). The median time to intervention was 23 h (17–32).

**Conclusion:**

Hemoglobin trends remained similar between groups until the morning of postoperative day 1. Clinical signs of bleeding did not develop until after the typical same day surgery observation period for most patients. These findings suggest that patients who develop clinically significant bleeding after same day bariatric surgery may not be identifiable prior to discharge and that next day discharge is a safer alternative.

**Key Points:**

•* Post-operative hemoglobin values after bariatric surgery were compared between patients who did or did not develop post-operative bleeding.*

•* Hemoglobin values were similar until the morning of post-operative day 1.*

•* The first clinical signs of bleeding developed at a median of 9 h after surgery.*

•* Some patients who develop clinically significant bleeding after same day bariatric surgery may not be identified prior to discharge.*

**Supplementary Information:**

The online version contains supplementary material available at https://doi.org/10.1007/s11695-026-08875-z.

## Introduction

Nearly 300,000 individuals undergo bariatric surgery in the United States each year [1]. Most patients are admitted after surgery, but the prevalence of same day bariatric surgery programs that discharge patients home on the day of surgery has risen to over 7% [2]. While establishment of same day programs was driven in part by the COVID-19 pandemic due to the inability to admit patients after elective operations [3–5], the cost savings associated with same day bariatric programs [6] also support ongoing efforts to optimize resource utilization and reduce costs within the American healthcare system [7, 8]. Several retrospective studies have shown same day bariatric surgery programs have similar morbidity and mortality through 30 days after surgery compared to patients who were admitted [9–11], further supporting the development of these programs.

Despite observational studies reporting similar outcomes between same day and inpatient bariatric surgery programs, there is limited data regarding bleeding incidence and outcomes for same day bariatric surgery programs. Two retrospective studies that utilized data from the Metabolic and Bariatric Surgery Accreditation and Quality Improvement Program (MBSAQIP) database concluded the incidence of bleeding and reoperation for 8,000 patients who underwent same day sleeve gastrectomy and 2,000 who underwent same day gastric bypass were similar to matched inpatient controls in the first 30 days after surgery [10, 11]. Another matched over 32,000 patients who underwent same day bariatric operations to patients who were admitted and found the same day group had lower rates of bleeding, reoperation, and readmissions [2]. While these studies suggest patients who undergo same day surgery are not at increased risk of post-operative bleeding, they lack data regarding the timing and treatment of clinically significant bleeding and the related patient outcomes.

To better understand the typical time course for the identification and treatment of post-operative bleeding after bariatric surgery, we conducted a retrospective observational study that aimed to characterize post-operative hemoglobin trends in patients who did or did not develop bleeding that required blood transfusion and/or surgical or endoscopic intervention. We hypothesized that hemoglobin trajectories would diverge within several hours of surgery.

## Methods

### Patient Selection

**Fig. 1:**
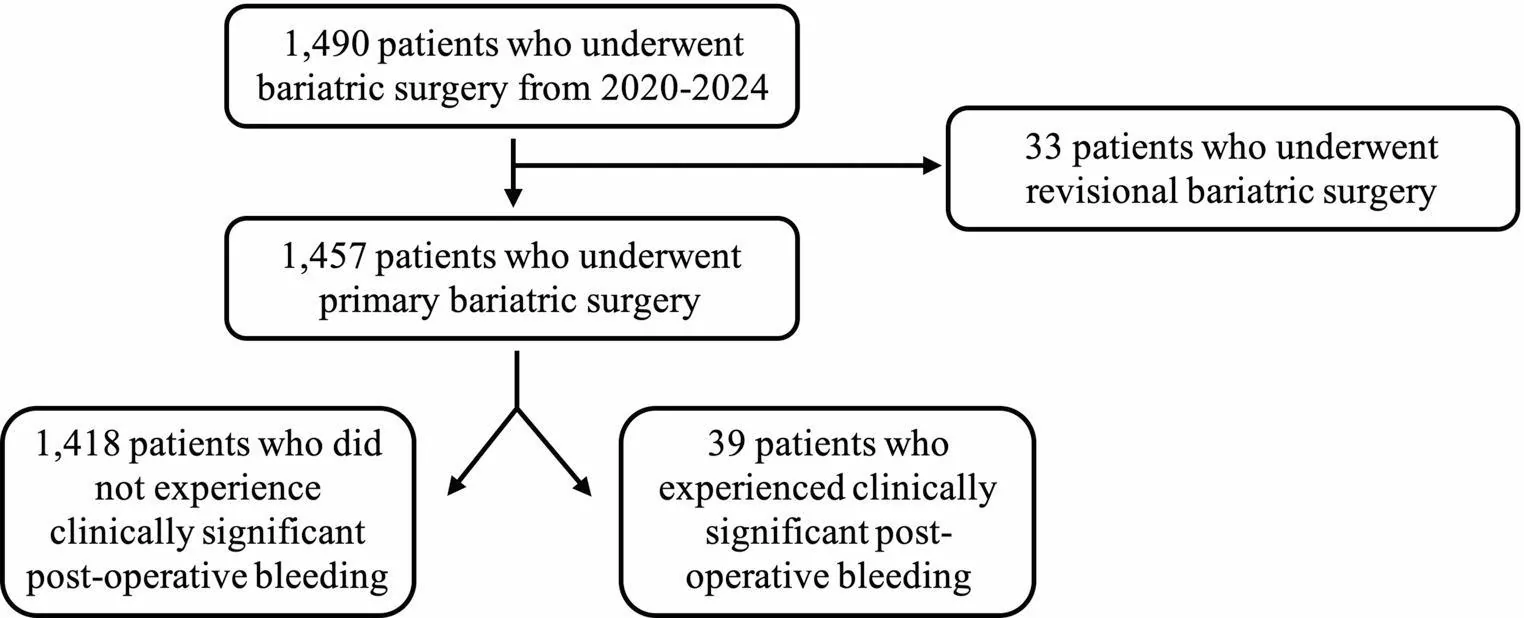
Strengthening the Report of Observational Studies in Epidemiology (STROBE) Diagram for patients who underwent bariatric surgery

Data from a single center’s prospectively maintained bariatric surgery (IRB #2014 − 0568) were utilized to conduct a retrospective analysis. Patients who underwent primary laparoscopic or robotic sleeve gastrectomy or Roux-en-Y gastric bypass between January 2020 and September 2024 were identified and stratified based on whether they developed clinically significant post-operative bleeding, defined as bleeding that required blood transfusion and/or surgical or endoscopic intervention within 30 days of surgery (Fig. 1). Patients undergoing revisional bariatric surgery were excluded.

### Study Variables

Patient characteristics were extracted from the bariatric surgery database as previously described [12, 13] and included patient age and body mass index (BMI) at the time of surgery, sex, race and ethnicity, bariatric operation (sleeve gastrectomy or Roux-en-Y gastric bypass), operative approach (laparoscopic or robotic), and receipt of intra- and post-operative venous thromboembolism (VTE) chemoprophylaxis. At our institution, intra-operative VTE prophylaxis consists of sequential compression devices and chemoprophylaxis. Most patients receive 40 or 60 mg of enoxaparin after induction of general anesthesia. Post-operatively, enoxaparin is initiated for continued VTE chemoprophylaxis either in the evening of post-operative day 0 or on the morning of post-operative day 1 based on attending surgeon preference and clinical stability. Post-operative chemoprophylaxis is typically held in the setting of suspected or confirmed bleeding. Medical conditions associated with obesity including gastroesophageal reflux disease, hyperlipidemia, hypertension, obstructive sleep apnea, and type II diabetes [14, 15] were abstracted using pre-operative patient problem lists, notes, and medication lists.

### Hemoglobin Trends

Peri-operative hemoglobin values and the timing of each draw in hours after the patient left the operating room were abstracted from the electronic health record. Patients undergoing bariatric surgery at our institution have three standardized hemoglobin draws, including pre-operatively within 30 days of surgery, four hours after surgery, and on the morning of post-operative day 1. Mean hemoglobin values for patients who did or did not bleed were calculated at each of these time points. The draw on the morning of post-operative day 1 occurred around 16 h after surgery for most patients. For patients with additional draws, the draw closest to 4–16 h was used. Average change in hemoglobin was also calculated between each of the three standardized draws.

### Outcomes

**Fig. 2:**
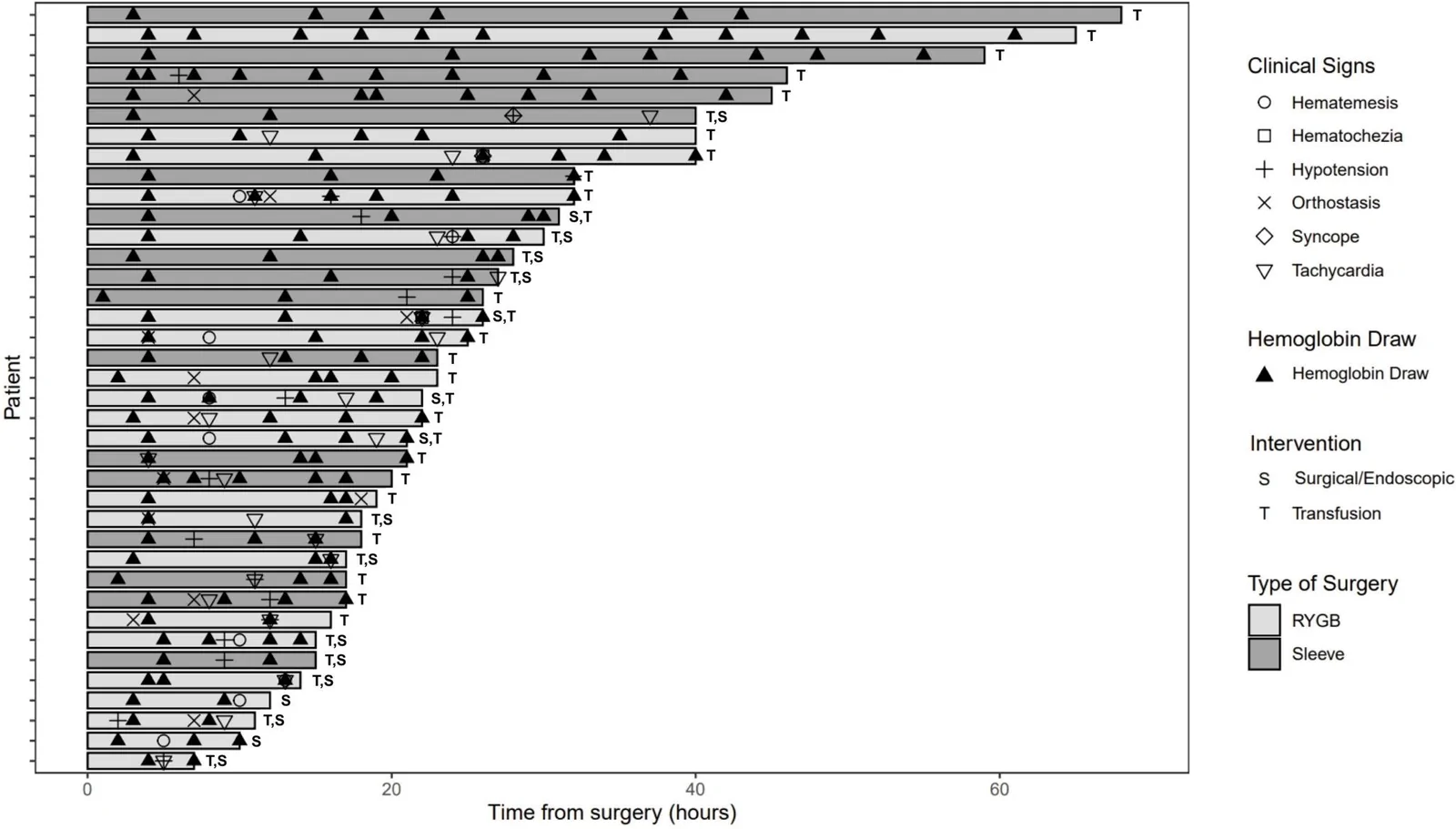
Timing of clinical events for patients who developed clinically significant bleeding. Swimmer plot for timing of hemoglobin draws and clinical signs of bleeding prior to transfusion and/or surgical or endoscopic intervention for 38 patients who developed clinically significant bleeding. Each bar represents a patient. Symbols represent the development of clinical signs, including hematemesis (circle), hematochezia (square), hypotension (cross), orthostasis (X), syncope (diamond), and tachycardia (inverted triangle). Hemoglobin draws are denoted by black triangles. The end of the bar represents the first intervention for bleeding, whether transfusion (T) or return to operating room for surgical or endoscopic intervention (S). For patients who required both transfusion and operative intervention, the first and second intervention are separated by a comma. Letters representing the first intervention appear after the bars for ease of interpretation. One patient who underwent a washout for an intra-abdominal hematoma 435 hours after surgery was excluded from this figure

The primary outcome was the mean change in hemoglobin between the pre-operative and four-hour post-operative draws for the patients who did or did not experience clinically significant bleeding. Secondary outcomes included the mean change in hemoglobin between protocolized lab draws, time to displaying clinical signs of bleeding, and time to intervention. Clinical signs included hypotension (systolic blood pressure < 90 mmHg), tachycardia (heart rate > 100), orthostasis (> 20 mmHg drop in systolic blood pressure or > 10 mmHg drop in diastolic blood pressure between back-to-back readings taken first while seated or lying followed by standing), syncope, hematemesis, and hematochezia. Time to first intervention was defined as the time in hours from leaving the operating room to when the first blood transfusion was initiated or when the patient entered the operating room for their surgical or endoscopic intervention, whichever occurred first. Median and interquartile range were utilized for time to clinical signs and intervention. One patient presented on post-operative day 17 with an intra-abdominal hematoma that required washout was excluded from Figs. 2 and S1 in order to better visualize the patient data. The number of units of blood transfused, bleeding source (gastrojejunal anastomosis, peri-gastric region, unknown, or other), length of stay, and readmission within 30 days were also documented.

### Sensitivity Analysis

As a sensitivity analysis, the primary analyses were repeated after stratification by operation (sleeve gastrectomy versus Roux-en-Y gastric bypass), BMI (< 40 kg/m² versus ≥ 40 kg/m²), and presence or absence of GERD. Within each stratum, hemoglobin values at the pre-operative, 4-hour post-operative, and post-operative day 1 time points were compared between patients who did or did not develop clinically significant bleeding to determine whether the timing of hemoglobin divergence differed across clinically relevant patient and operative subgroups. The non-parametric Wilcoxon rank sum test for hypothesis testing was used to compare values given the small size of some of the subgroups.

### Statistical Analyses

All analyses were performed with SAS software (version 9.4, SAS Institute Inc., Cary, NC). R statistical software (version 4.5.1) was used for figure development. *P*-values were generated using chi-squared tests for categorical variables and two-tailed unpaired T-tests for continuous variables. The Strengthening the Report of Observational Studies in Epidemiology (STROBE) guidelines for case-control studies were reviewed and adhered to [16].

## Results

### Patient Characteristics

A total of 1,457 patients were included in the study with characteristics as described in Table 1. Thirty-nine patients (2.7%) experienced clinically significant bleeding. Patients across the groups had similar mean age (47.0 years [SD 10.5] vs. 45.4 years [SD 10.8]; *p* = 0.351). Most were female (82.1% vs. 82.0%, *p* > 0.999) and White, non-Hispanic (87.2% vs. 85.4%; *p* = 0.830). Mean BMI was lower for patients who bled (42.9 kg/m^2^ [SD 6.0] vs. 45.9 kg/m^2^ [SD 7.1]; *p* = 0.008). Patients who bled were more likely to have a diagnosis of gastroesophageal reflux disease (66.7% vs. 49.0%; *p* = 0.034) and hyperlipidemia (46.2% vs. 28.6%; *p* = 0.030). More patients underwent sleeve gastrectomy (60.6%: 52.3% laparoscopic, 8.3% robotic) compared to Roux-en-Y gastric bypass (39.4%: 34.5% laparoscopic, 4.9% robotic). Roux-en-Y gastric bypass was more common in those who bled (56.4%). Most patients (98.4%) received intra-operative VTE chemoprophylaxis. The number of patients who received VTE chemoprophylaxis on post-operative day 1 was lower for those who bled compared to those who did not (59% vs. 95.8%), as chemoprophylaxis was typically held after bleeding was recognized.

**Table 1:** Characteristics for patients undergoing bariatric surgery who did or did not experience clinically significant post-operative bleeding

Variables	Total *n* = 1,457	Clinically Significant Bleeding		
		Yes *n* = 39	No *n* = 1,418	*P*-Value
Age in years, Mean (SD)	45.4 (10.8)	47.0 (10.5)	45.4 (10.8)	0.351
Sex at Birth, n (%)				> 0.999
Male	262 (18.0)	7 (18.0)	255 (18.0)	
Female	1,195 (82.0)	32 (82.1)	1,163 (82.0)	
Race/Ethnicity, n (%)				0.830
White, non-Hispanic	1,245 (85.5)	34 (87.2)	1,211 (85.4)	
Black, non-Hispanic	99 (6.8)	4 (10.3)	95 (6.7)	
Hispanic	78 (5.4)	1 (2.6)	77 (5.4)	
Asian, non-Hispanic	7 (0.5)	0 (0.00)	7 (0.5)	
Other	18 (0.7)	0 (0.00)	18 (1.3)	
Unknown	10 (0.7)	0 (0.00)	10 (0.7)	
BMI (kg/m^2^), Mean (SD)	45.8 (7.1)	42.9 (6.0)	45.9 (7.1)	0.008
Pre-Operative Associated Medical Problems, n (%)				
Gastroesophageal Reflux Disease	721 (49.5)	26 (66.7)	695 (49.0)	0.034
Hyperlipidemia	424 (29.1)	18 (46.2)	406 (28.6)	0.030
Hypertension	751 (51.5)	23 (59.0)	728 (51.3)	0.418
Obstructive Sleep Apnea	1,038 (71.2)	28 (71.8)	1,010 (71.2)	> 0.999
Type 2 Diabetes Mellitus	389 (26.7)	13 (33.3)	376 (26.5)	0.360
Bariatric Operation, n (%)				0.031
Sleeve Gastrectomy	883 (60.6)	17 (43.6)	866 (61.1)	
Roux-en-Y Gastric Bypass	574 (39.4)	22 (56.4)	552 (38.9)	
Received Intra-Operative Chemoprophylaxis, n (%)	1,433 (98.4)	38 (97.4)	1,395 (98.4)	0.481
Received Post-Operative Day 1 Chemoprophylaxis, n (%)	1,382 (94.9)	23 (59.0)	1,359 (95.8)	< 0.0001
Operation Time ≥3 h, n (%)	88 (6.0)	2 (5.1)	86 (6.1)	> 0.999

### Characteristics and Outcomes of Patients who Bled

Clinical characteristics and outcomes for the 39 patients who experienced clinically significant bleeding are displayed in Table 2. Seventeen (43.6%) patients underwent sleeve gastrectomy, and 22 (56.4%) underwent Rou-en-Y gastric bypass. Buttressed staples were used for most sleeve gastrectomies (70.6%). The gastrojejunal anastomosis was created using an end-to-end anastomosis circular stapler (77.3%) more frequently than a linear stapler (22.7%). Prior to admission, 2 (5.1%) patients were anti-coagulated and 7 (17.9%) were taking 81 mg of aspirin.

**Table 2:** Characteristics of patients undergoing bariatric surgery who experienced clinically significant bleeding

Variable	All *n* = 39	Sleeve Gastrectomy *n* = 17	Roux-en-Y Gastric Bypass *n* = 22
Clinical Signs and Symptoms, *n* (%)			
Hypotension	18 (46.2)	11 (64.7)	7 (31.8)
Tachycardia	24 (61.5)	8 (47.1)	16 (72.7)
Orthostasis	13 (33.3)	3 (17.6)	10 (45.5)
Syncope	2 (5.1)	1 (5.9)	1 (4.5)
Hematemesis	11 (28.2)	0 (0.0)	11 (50.0)
Hematochezia	5 (12.8)	0 (0.0)	5 (22.7)
Time to First Clinical Sign or Symptom in Hours, Median (IQR)	9 (6.25–17.5)	10 (7-20.25)	9 (5–16)
Prior to Admission Medications, n (%)			
Anti-Coagulation	2 (5.1)	1 (5.9)	1 (4.5)
Anti-Platelet Therapy	7 (17.9)	4 (23.5)	3 (13.6)
Timing of Post-Operative Intervention, n (%)			
Post-Operative Day 0	9 (23.1)	4 (23.5)	5 (22.7)
Post-Operative Day 1	24 (61.5)	9 (52.9)	15 (68.2)
Post-Operative Day 2	4 (10.3)	2 (11.8)	2 (9.1)
Post-Operative Day *≥* 3	2 (5.1)	1 (5.9)	1 (4.5)
Time to First Intervention, Median (IQR)	23 (17–32)	27 (20–40)	21.5 (15.25-29)
Received Blood Transfusion, n (%)	36 (92.3)	17 (100.0)	19 (86.4)
Number of Units Transfused, Median (IQR)	2 (1–3)	2 (1–3)	2 (1.5–3.5)
Required Intervention, n (%)	18 (46.2)	5 (29.4)	13 (59.1)
Esophagoduodenoscopy with Clipping	8 (20.5)	0 (0.0)	8 (36.4)
Diagnostic Laparoscopy	10 (25.6)	5 (29.4)	5 (22.7)
Source of Bleeding, n (%)			
Gastrojejunal Anastomosis	11 (28.2)	N/A	11 (50.0)
Peri-gastric	7 (17.9)	5 (29.4)	2 (9.1)
Other	10 (25.6)	7 (41.2)	3 (13.6)
Unknown	11 (28.2)	5 (29.4)	6 (27.3)
Length of Stay in days, Mean (SD)	3.3 (2.2)	3.1 (1.2)	3.3 (2.2)
Readmitted within 90 Days, n (%)	9 (23.1)	6 (35.3)	3 (13.6)

Clinical signs and symptoms include those that developed prior to when patients were first transfused or taken back to the operating room. Timing of post-operative intervention was defined as the post-operative date of the first intervention. Source of bleeding was defined based on post-operative imaging or intra-operative findings. Patients with unknown sources were transfused but did not undergo any additional diagnostic work up. Peri-gastric bleeds included any blood products in the left upper quadrant

Many patients who bled had accompanying clinical signs and symptoms, including tachycardia (61.5%) and hypotension (46.2%). Most patients who bled after sleeve gastrectomy became hypotensive (64.7%), while most patients who underwent Roux-en-Y gastric bypass and bled developed tachycardia (72.7%). Post-operative day 1 was the most common time for intervention (61.5%). The median number of units of blood received by the 36 patients who were transfused was 2 (IQR 1–3). Surgical or endoscopic intervention was required by 18 patients (46.2%), with 10 undergoing diagnostic laparoscopy and 8 treated with esophagoduodenoscopy with clipping.

The clinical timeline for the patients who bled, including development of clinical signs of bleeding and timing of hemoglobin draws, is depicted in Fig. 2. The first hemoglobin draw for most patients was around 4 h after surgery, after which the timing and frequency of hemoglobin draws varied between patients. The median number of draws prior to transfusion and/or intervention was 4 (IQR 3–5; min 2, max 11) compared to 2 (IQR 2–2; min 1, max 19) for those who did not bleed. Clinical signs of bleeding were developed by 34 (87.2%) patients. The median time from surgery to the first clinical sign of bleeding was 9 h (IQR 6.25–17.5; min 2, max 396). The median time to intervention was 23 h (IQR 17–32, min 7, max 435). Patients with longer times to intervention displayed fewer clinical signs and were predominately transfused compared to those with shorter times to intervention who displayed multiple clinical signs and often underwent surgical intervention.

### Hemoglobin Trends

Mean hemoglobin values are described in Table 2. The mean pre-operative and four-hour post-operative hemoglobin values were similar between groups. Patients who developed post-operative bleeding had a mean hemoglobin value almost two points lower than those who did not bleed by the morning of post-operative day 1 [10.7 g/dL (SD 1.6) bleed vs. 12.5 g/dL (SD 1.1) no bleed; *p* < 0.001].

**Fig. 3:**
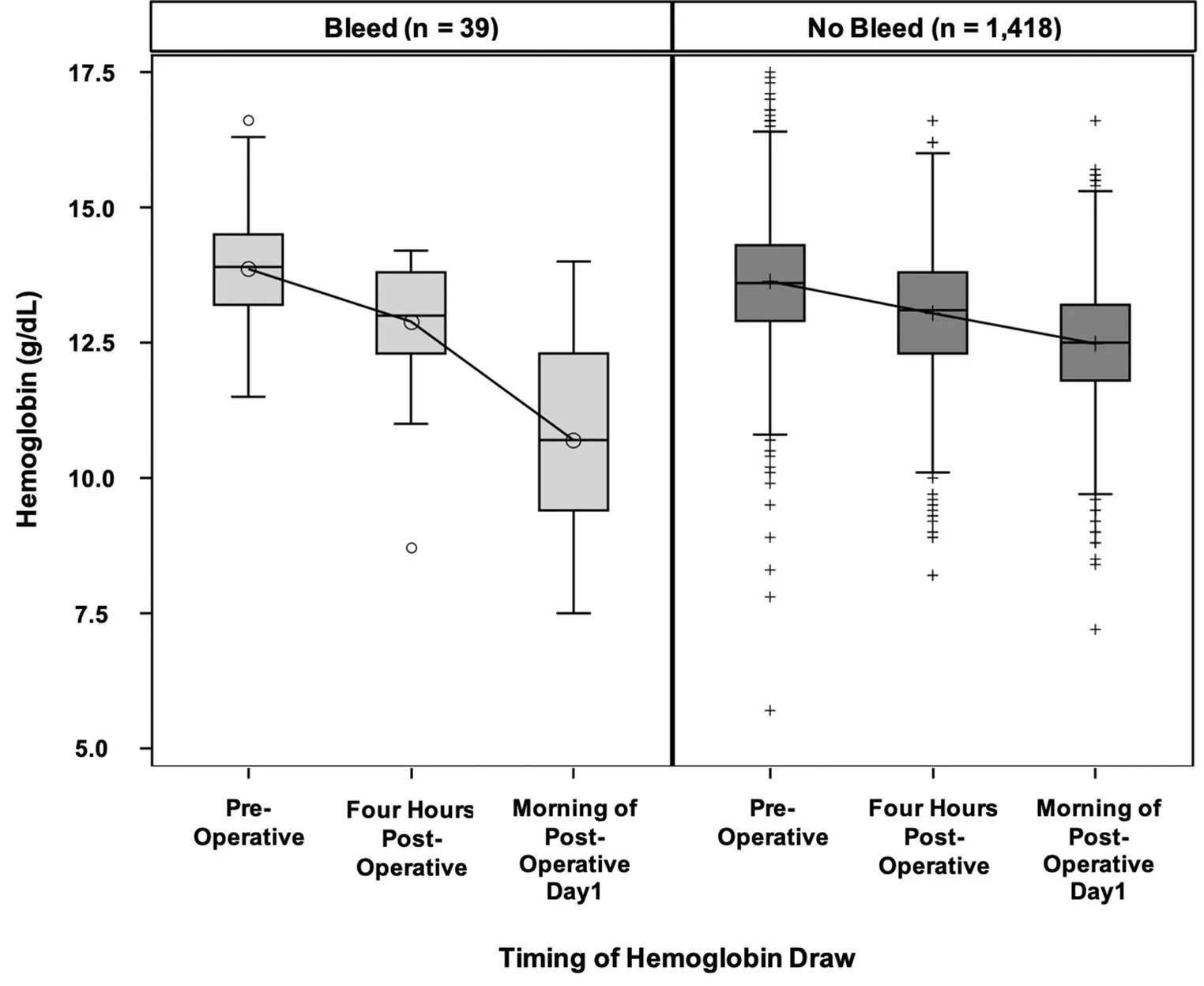
Peri-operative hemoglobin trends for patients undergoing bariatric surgery who did or did not experience clinically significant post-operative bleeding. Trends in hemoglobin values (g/dL) for patients who developed clinically significant bleeding (left panel, light gray) and those who did not bleed (right panel, dark gray) at three protocolized lab draws (pre-operatively, four hours post-operatively, and morning of post-operative day 1). The trend lines depicts the mean hemoglobin value at each time point. The box represents the median and interquartile range (IQR). The whiskers depict the min and max or 1.5 times the IQR for time points with individual hemoglobin values that fall outside 1.5 times the IQR (denoted by individual circles or crosses)

The mean changes in hemoglobin between pre-operative, four-hour post-operative, and morning of post-operative day 1 draws are displayed in Table 3; Fig. 3, while individual hemoglobin trends for each patient who bled are depicted in Figure S1. Patients who bled had a mean change in hemoglobin of -1.0 g/dL (SD 1.3) from the pre-operative draw to four hours post-operatively compared to -0.6 g/dL (SD 0.9) for those who did not bleed (*p* = 0.068). The difference in hemoglobin values between four hours post-operatively and the morning of post-operative day 1 was − 2.2 g/dL (SD 1.3) for those with bleeding versus − 0.6 g/dL (SD 0.8) for those without (*p* < 0.0001).

**Table 3:** Mean change in hemoglobin for patients undergoing bariatric surgery who did or did not experience clinically significant post-operative bleeding

Variable	Bleed *n* = 39	No Bleed *n* = 1,418	*P*-Value
Hemoglobin (g/dL), Mean (SD)			
Pre-Operative	13.9 (1.2)	13.6 (1.2)	0.232
Four-Hour Post-Operative	12.9 (1.1)	13.0 (1.2)	0.427
Morning of Post-Operative Day 1	10.7 (1.6)	12.5 (1.1)	< 0.0001
Change in Hemoglobin, Mean (SD)			
Pre-Operative and Four-Hour Post-Operative	-1.0 (1.3)	-0.6 (0.9)	0.068
Four-Hour Post-Operative and Morning of Post-Operative Day 1	-2.2 (1.3)	-0.6 (0.8)	< 0.0001
Pre-Operative and Morning of Post-Operative Day 1	-3.2 (1.8)	-1.2 (0.9)	< 0.0001

Mean hemoglobin values in g/dL at time points for each of the three protocolized lab draws (pre-operatively, four hours post-operatively, and morning of post-operative day 1). The morning of post-operative day 1 draw was taken around 16 h post-operatively for most patients. For patients with additional lab draws, the values closest to 4 and 16 h were used

The overall pattern of change in hemoglobin was unchanged when stratified by operation type, BMI, and GERD (Tables S1-S3). Within each subgroup, hemoglobin values remained similar at the 4-hour post-operative assessment and did not diverge until the morning of post-operative day 1 between patients who did or did not develop clinically significant bleeding.

## Discussion

Our study aimed to characterize the trends in hemoglobin values between patients who do or do not develop clinically significant bleeding after primary bariatric surgery. We found that hemoglobin trends were similar four hours after surgery but diverged by the morning of postoperative day 1, a pattern that was consistent across clinically relevant patient and operative subgroups. The first clinical signs of bleeding typically developed nearly 10 h after surgery, while the median time to intervention was just under 24 h.

Mean hemoglobin values for patients who did or did not bleed were similar at four hours after surgery before diverging on the morning of post-operative day 1. This suggests that the patient condition between 4 and 16 h after surgery is important for identifying post-operative bleeding. This is relevant in the setting of same day bariatric surgery, where patients are often observed for around six hours after surgery [17–20]. While discharge criteria vary between programs, patients typically need to be normotensive with a heart rate < 100 and have adequate pain control, tolerate clear liquids, ambulate, and void prior to discharge [17–21]. Three studies utilized post-operative hemoglobin to assess for bleeding. One used a cutoff of a decrease of < 2 g/dL at four hours after surgery [22] while two others applied a cutoff of < 1 mmol/L (1.61 g/dL) at six hours after surgery [19, 20]. A total of 5 out of 1,025 (0.5%) patients from those studies were admitted after surgery due to a drop in hemoglobin greater than the discharge criteria, accounting for a third of the patients with confirmed post-operative bleeding. In our cohort, patients who bled had an average change in hemoglobin between the pre-operative and four-hour post-operative draws of 1.0 g/dL, meaning more than half would have met the hemoglobin cut offs for discharge. Considering the similarity and magnitude of change in four-hour post-operative hemoglobin values between those who did or did not bleed in our study in conjunction with the low number of same day bariatric patients admitted based on their hemoglobin value in recovery, the ability of four-hour post-operative hemoglobin to identify post-operative bleeding is limited. This may reflect post-operative hemodilution from intravenous resuscitation, as well as the inherent delay between the onset of bleeding and the corresponding drop in hemoglobin on laboratory tests. As a result, the utility of hemoglobin values is highest when interpreted along with clinical signs of bleeding.

Patients who developed post-operative bleeding first displayed clinical signs of bleeding at a median of 9 h after surgery, which often preceded a change in hemoglobin. While hypotension, tachycardia, hematemesis, and hematochezia are known signs of post-operative bleeding [23, 24], few studies have investigated when these symptoms develop in bariatric surgery patients. One retrospective study that characterized changes in vital signs for 20 patients who bled after undergoing laparoscopic gastric bypass found tachycardia was the first sign of bleeding and was noted on average 7 h after surgery [25], similar to the median in our study. A randomized clinical trial comparing outcomes of nearly 1,500 patients who underwent same day versus inpatient sleeve gastrectomy reported four same day patients developed tachycardia prior to discharge and were admitted for observation, while two inpatients became tachycardic the night after surgery with one requiring a blood transfusion [17]. A retrospective study of 500 patients who underwent same day gastric bypass noted seven patients were admitted from recovery due to abnormal vital signs [19]. Twenty-one were discharged and then readmitted within 48 h of surgery. Nearly half had developed hematemesis or hematochezia, although none required transfusion or intervention. A Dutch study that retrospectively reviewed outcomes from 260 patients who underwent same day sleeve gastrectomy or gastric bypass admitted one patient after surgery due tachycardia and one with hematochezia treated with tranexamic acid. Eight patients (3%) were readmitted within 48 h with six who developed hematochezia and one who had a syncopal episode with an associated drop in hemoglobin [20]. Taken together, these studies suggest that while some patients develop clinical signs of bleeding shortly after surgery, many are asymptomatic prior to same day surgery discharge and ultimately represent, consistent with the timeline identified in our study.

The median time to intervention for clinically significant bleeding was just under 24 h. This pattern has been noted by several studies. A single center retrospective analysis of 359 patients who required reoperation after bariatric surgery, most commonly for hemorrhage control, found 32% of those who underwent sleeve gastrectomy and 24% of those who underwent gastric bypass were intervened upon within 24 h of their index operation [26]. Similarly, a retrospective study of 39 patients who experienced bleeding that required intervention or transfusion after undergoing sleeve gastrectomy or gastric bypass concluded 75% were diagnosed within 48 h of surgery and 92% underwent intervention within 24 h of detection [27]. Several single center studies detailing outcomes after same day bariatric surgery described interventions within 24 h for post-operative bleeding. A retrospective study of over 2,500 patients who underwent sleeve gastrectomy at an ambulatory surgery center with the ability to observe patients for up to 23 h after surgery found 31 patients were transferred to a hospital for further management [28]. Bleeding was the most common reason for transfer and accounted for 15 of 35 reoperations, although timing of the reoperations was not specified. In another study of 265 same day bariatric operations, one patient was readmitted within 24 h for bleeding from the gastric sleeve staple line that required transfusion [22]. These studies demonstrate that intervention for post-operative bleeding after bariatric surgery consistently occurs within 24 h of surgery, including in patients who underwent same day bariatric surgery and required readmission.

Even though most patients who undergo bariatric surgery do not develop post-operative bleeding, the associated increased morbidity and mortality of bleeding [29, 30]. must be accounted for and minimized irrespective of practice model. The reported incidence of bleeding after bariatric surgery ranges from 0.4%-4.4% [31–33] and varies by type of operation, operative approach, and the definition of bleeding. The incidence of bleeding in our study was at the middle to upper end of the reported range at 2.7%. This likely reflects the inclusion of patients who underwent both sleeve gastrectomy and gastric bypass, which is known to have increased risk of bleeding [31, 34, 35], and our definition of bleeding that included any transfusion within 30 days of surgery. One potential way to decrease the risk of post-operative bleeding is by administering prophylactic tranexamic acid intra-operatively. This has been associated with smaller drops in post-operative hemoglobin, fewer bleeding and transfusion events, and shorter lengths of stay with no increase in the incidence of thromboembolic events [36, 37].

Our findings suggest that most patients who develop post-operative bleeding after undergoing bariatric surgery do not display clinical or laboratory signs of bleeding until after they would have been discharged following same day bariatric surgery. These findings support next-day discharge as a potentially safer alternative because it accommodates overnight observation while still allowing most patients to discharge within 24 h of surgery. This additional observation period may facilitate earlier identification of post-operative bleeding and reduce delays in treatment, emergency department visits, and readmissions [38, 39]. Additionally, the facility resources where the index operation is performed such as imaging and laboratory services, operating room and endoscopy availability, and the ability to admit patients after surgery must be considered, as patients who develop post-operative bleeding at surgery centers lacking these capabilities must be transferred to higher levels of care and could experience delays in treatment.

Of note, all patients in this study were admitted after undergoing bariatric surgery. Same day bariatric surgery protocols typically select for “low risk” younger patients with lower BMIs and fewer associated medical problems [21, 40–42]. Given the patients in our cohort had similar patient characteristics including age, BMI, and type of operation compared to those included in same day studies, the timeline for bleeding after bariatric surgery described in this paper remains relevant to the same day setting. However, further investigation comparing outcomes for those who develop clinically significant bleeding after same day versus inpatient surgery is warranted, including time to diagnosis and intervention, length of stay, need for readmission, and cost.

This study has several limitations. First, the number of patients who bled was relatively small. A cohort with a larger number of bleeds may have differing trends that could be stratified by patient characteristics or operation type. Second, while there was a trend towards patients who bled experiencing a larger change in hemoglobin at four hours after surgery, there was no statistically significant difference. This could become significant in a larger cohort. Third, our study defined post-operative bleeding based on the need for transfusion and/or intervention in order to ensure bleeding was consistently and objectively identified. Patients who were suspected of bleeding and managed with clinical observation alone were not identified and may have exhibited different clinical patterns than those reported in this study. Lastly, as a retrospective observational study, our analyses could be limited by unmeasured confounding such as intra-operative blood loss or volume of intravenous resuscitation.

## Conclusion

Hemoglobin trends remained similar between patients who did or did not bleed until the morning of postoperative day 1. Most patients who bled developed clinical signs of bleeding after the typical six-hour same day surgery observation period. These findings suggest that four-hour post-operative hemoglobin values and short post-operative observation periods are inadequate for identifying bleeding after bariatric surgery. Bleeding is more effectively identified during overnight observation, which better allows for identification of changes in vital signs and decreases in hemoglobin.

Timing of hemoglobin draws and clinical signs of bleeding prior to transfusion and/or surgical or endoscopic intervention for 38 patients who developed clinically significant bleeding. Each bar represents a patient. Symbols represent the development of clinical signs, including hematemesis (circle), hematochezia (square), hypotension (cross), orthostasis (X), syncope (diamond), and tachycardia (inverted triangle). Hemoglobin draws are denoted by black triangles. The end of the bar represents the first intervention for bleeding, whether transfusion (T) or return to operating room for surgical or endoscopic intervention (S). For patients who required both transfusion and operative intervention, the first and second intervention are separated by a comma. Letters representing interventions appear after the bars for ease of interpretation. One patient who underwent a washout for an intra-abdominal hematoma 435 h after surgery was excluded from this figure.

Trends in hemoglobin values (g/dL) for patients who developed clinically significant bleeding (left panel, light gray) and those who did not bleed (right panel, dark gray) at three protocolized lab draws (pre-operatively, four hours post-operatively, and morning of post-operative day 1). The trend lines depicts the mean hemoglobin value at each time point. The box represents the median and interquartile range (IQR). The whiskers depict the min and max or 1.5 times the IQR for time points with individual hemoglobin values that fall outside 1.5 times the IQR (denoted by individual circles or crosses).

## Supplementary Information

Below is the link to the electronic supplementary material.

Supplementary Material 1

## Data Availability

The data that support the findings of this study are available from the corresponding author upon reasonable request.
