# Supplementary material for: Post-operative Hemoglobin Trends for Patients Undergoing Bariatric Surgery: A Retrospective Observational Study
Source: Obes Surg. 2026 Jul 25;36(9):4791–800. doi: 10.1007/s11695-026-08875-z (PMC13562350; doi:10.1007/s11695-026-08875-z)
Supplement: Supplementary file 1 [file 11695_2026_8875_MOESM1_ESM.docx]

**Figure S1:** **Hemoglobin trends for patients who developed clinically significant bleeding**

Trends in hemoglobin values prior to first transfusion or surgical/endoscopic intervention for 38 patients who developed clinically significant bleeding. Each line represents an individual patient. Length of lines varies based on the time after surgery to first intervention. One patient who underwent a washout for an intra-abdominal hematoma 435 hours after surgery was excluded from this figure.

**Table S1: Change in hemoglobin stratified by operation type**

| **Variable** | **Sleeve Gastrectomy** | | | **Roux-en-Y**  **Gastric Bypass** | | |
| --- | --- | --- | --- | --- | --- | --- |
|  | **Bleed**  **n = 17** | **No Bleed**  **n = 866** | **P-Value** | **Bleed**  **n = 22** | **No Bleed**  **n = 552** | **P-Value** |
| **Hemoglobin (g/dL), Mean (SD)** |  |  |  |  |  |  |
| Pre-Operative | 13.9 (1.2) | 13.6 (1.2) | 0.279 | 13.8 (1.1) | 13.7 (1.1) | 0.565 |
| Four-Hour Post-Operative | 13.2 (0.7) | 13.1 (1.2) | 0.739 | 12.7 (1.3) | 13.0 (1.2) | 0.408 |
| Morning of Post-Operative Day 1 | 10.9 (1.7) | 12.6 (1.2) | <0.001 | 10.5 (1.6) | 12.3 (1.1) | <0.0001 |
| **Change in Hemoglobin, Mean (SD)** |  |  |  |  |  |  |
| Pre-Operative and Four-Hour Post-Operative | -0.8 (1.4) | -0.6 (0.8) | 0.944 | -1.1 (1.1) | -0.6 (1.0) | 0.073 |
| Four-Hour Post-Operative and Morning of Post-Operative Day 1 | -2.2 (1.4) | -0.5 (0.7) | <0.0001 | -2.2 (1.2) | -0.7 (0.8) | <0.0001 |
| Pre-Operative and Morning of Post-Operative Day 1 | -3.0 (2.3) | -1.0 (0.8) | <0.001 | -3.3 (1.4) | -1.3 (1.0) | <0.0001 |

Mean hemoglobin values at three standardized time points (pre-operatively, four hours post-operatively, and morning of post-operative day 1) and mean change in hemoglobin between time points stratified by operation type (sleeve gastrectomy, Roux-en-Y gastric bypass). Hemoglobin values did not diverge between patients who did or did not bleed until the morning of post-operative day 1 for both patients who underwent Roux-en-Y gastric bypass and sleeve gastrectomy. The morning of post-operative day 1 draw was taken around 16 hours post-operatively for most patients. For patients with additional lab draws, the values closest to 4 and 16 hours were used.

**Table S2: Change in hemoglobin stratified by pre-operative body mass index**

| **Variable** | **BMI <40 kg/m^2^** | | | **BMI ≥40 kg/m^2^** | | |
| --- | --- | --- | --- | --- | --- | --- |
|  | **Bleed**  **n = 13** | **No Bleed**  **n = 301** | **P-Value** | **Bleed**  **n = 26** | **No Bleed**  **n = 1,117** | **P-Value** |
| **Hemoglobin (g/dL), Mean (SD)** |  |  |  |  |  |  |
| Pre-Operative | 14.1 (1.2) | 13.7 (1.1) | 0.263 | 13.8 (1.1) | 13.6 (1.2) | 0.600 |
| Four-Hour Post-Operative | 12.7 (1.4) | 13.0 (1.1) | 0.783 | 13.0 (1.0) | 13.1 (1.2) | 0.824 |
| Morning of Post-Operative Day 1 | 10.1 (1.8) | 12.4 (1.1) | <0.0001 | 11.0 (1.5) | 12.5 (1.1) | <0.0001 |
| **Change in Hemoglobin, Mean (SD)** |  |  |  |  |  |  |
| Pre-Operative and Four-Hour Post-Operative | -1.4 (1.3) | -0.7 (0.8) | 0.072 | -0.8 (1.2) | -0.6 (0.9) | 0.704 |
| Four-Hour Post-Operative and Morning of Post-Operative Day 1 | -2.6 (1.7) | -0.6 (0.8) | <0.001 | -2.0 (1.0) | -0.6 (0.8) | <0.0001 |
| Pre-Operative and Morning of Post-Operative Day 1 | -3.9 (2.0) | -1.3 (0.9) | <0.0001 | -2.8 (1.6) | -1.1 (0.9) | <0.0001 |

Mean hemoglobin values at three standardized time points (pre-operatively, four hours post-operatively, and morning of post-operative day 1) and mean change in hemoglobin between time points stratified by pre-operative body mass index (BMI) (<40 kg/m^2^, ≥40 kg/m^2^). Hemoglobin values did not diverge between patients who did or did not bleed until the morning of post-operative day 1 for both patients with a BMI of <40 kg/m^2^ and ≥40 kg/m^2^. The morning of post-operative day 1 draw was taken around 16 hours post-operatively for most patients. For patients with additional lab draws, the values closest to 4 and 16 hours were used.

**Table S3: Change in hemoglobin stratified by presence or absence of pre-operative GERD**

| **Variable** | **Pre-Operative GERD** | | | **No Pre-Operative GERD** | | |
| --- | --- | --- | --- | --- | --- | --- |
|  | **Bleed**  **n = 26** | **No Bleed**  **n = 723** | **P-Value** | **Bleed**  **n = 13** | **No Bleed**  **n = 695** | **P-Value** |
| **Hemoglobin (g/dL), Mean (SD)** |  |  |  |  |  |  |
| Pre-Operative | 13.7 (1.0) | 13.6 (1.2) | 0.803 | 14.3 (1.4) | 13.7 (1.2) | 0.092 |
| Four-Hour Post-Operative | 12.8 (1.2) | 13.0 (1.2) | 0.643 | 13.1 (0.8) | 13.1 (1.2) | 0.991 |
| Morning of Post-Operative Day 1 | 10.5 (1.8) | 12.4 (1.1) | <0.0001 | 11.0 (1.4) | 12.6 (1.1) | <0.001 |
| **Change in Hemoglobin, Mean (SD)** |  |  |  |  |  |  |
| Pre-Operative and Four-Hour Post-Operative | -0.9 (1.1) | -0.6 (0.9) | 0.472 | -1.2 (1.6) | -0.6 (1.0) | 0.150 |
| Four-Hour Post-Operative and Morning of Post-Operative Day 1 | -2.3 (1.3) | -0.6 (0.9) | <0.0001 | -2.1 (1.3) | -0.5 (0.8) | <0.0001 |
| Pre-Operative and Morning of Post-Operative Day 1 | -3.1 (1.8) | -1.2 (0.9) | <0.0001 | -3.2 (1.9) | -1.1 (0.9) | <0.0001 |

Mean hemoglobin values at three standardized time points (pre-operatively, four hours post-operatively, and morning of post-operative day 1) and mean change in hemoglobin between time points stratified by presence or absence of pre-operative gastroesophageal reflux disease (GERD). Hemoglobin values did not diverge between patients who did or did not bleed until the morning of post-operative day 1 for both patients with and without GERD. The morning of post-operative day 1 draw was taken around 16 hours post-operatively for most patients. For patients with additional lab draws, the values closest to 4 and 16 hours were used.
